# A Direct Assay for Measuring the Activity and Inhibition of Coactivator-Associated Arginine Methyltransferase 1

**DOI:** 10.1021/acs.biochem.2c00075

**Published:** 2022-05-17

**Authors:** Yurui Zhang, Matthijs J. van Haren, Nils Marechal, Nathalie Troffer-Charlier, Vincent Cura, Jean Cavarelli, Nathaniel I. Martin

**Affiliations:** † Biological Chemistry Group, Institute of Biology Leiden, Leiden University, Sylviusweg 72, 2333 BE Leiden, The Netherlands; ‡ Department of Integrated Structural Biology, Institut de Génétique et de Biologie Moléculaire et Cellulaire, CNRS UMR 7104, INSERM U 1258, Université de Strasbourg, Illkirch F-67404, France

## Abstract

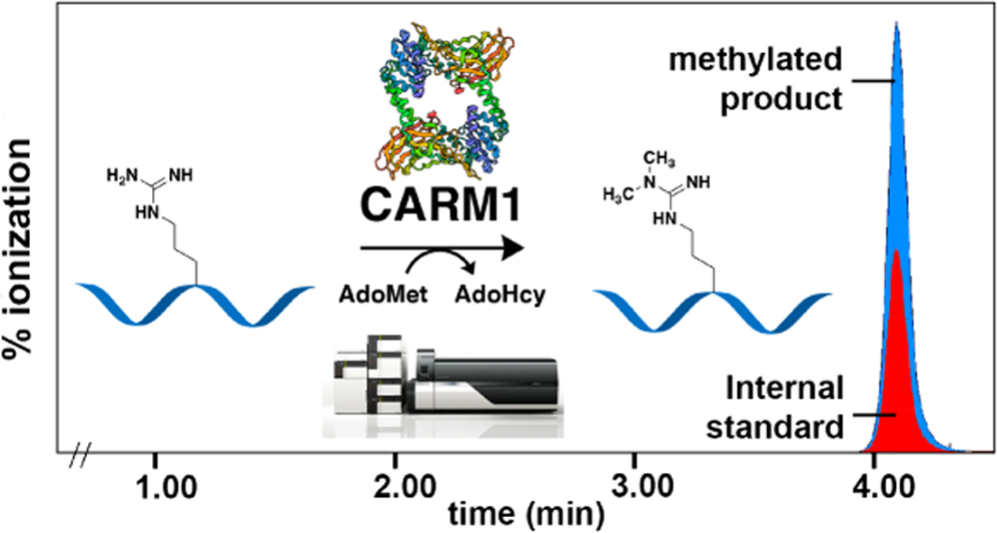

Coactivator-associated arginine methyltransferase 1 (CARM1) is a member of the family of protein arginine methyltransferases. CARM1 catalyzes methyl group transfer from the cofactor *S*-adenosyl-l-methionine (AdoMet) to both histone and nonhistone protein substrates. CARM1 is involved in a range of cellular processes, mainly involving RNA transcription and gene regulation. As the aberrant expression of CARM1 has been linked to tumorigenesis, the enzyme is a potential therapeutic target, leading to the development of inhibitors and tool compounds engaging with CARM1. To evaluate the effects of these compounds on the activity of CARM1, sensitive and specific analytical methods are needed. While different methods are currently available to assess the activity of methyltransferases, these assays mainly focus on either the measurement of the cofactor product *S*-adenosyl-l-homocysteine (AdoHcy) or employ radioactive or expensive reagents, each with their own advantages and limitations. To complement the tools currently available for the analysis of CARM1 activity, we here describe the development of a convenient assay employing peptide substrates derived from poly(A)-binding protein 1 (PABP1). This operationally straightforward liquid chromatography-tandem mass spectrometry (LC-MS/MS)-based approach allows for the direct detection of substrate methylation with minimal workup. The method was validated, and its value in characterizing CARM1 activity and inhibition was demonstrated through a comparative analysis involving a set of established small molecules and peptide-based CARM1 inhibitors.

## Introduction

**Figure 1:**
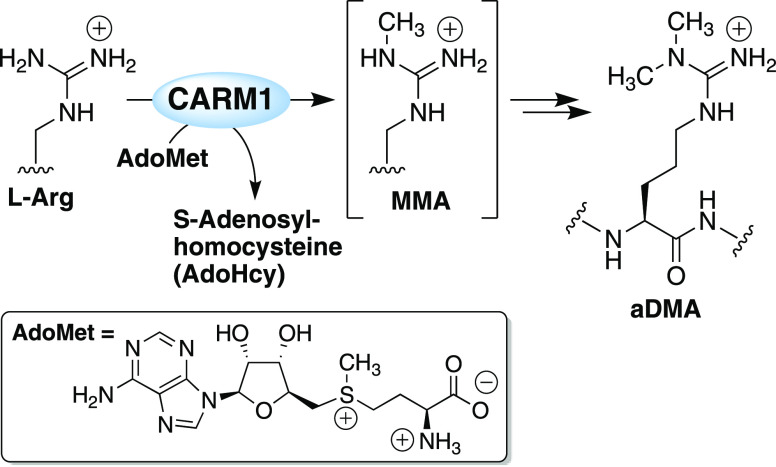
CARM1 catalyzes the methylation of arginine residues in substrate proteins and peptides to generate monomethyl arginine (MMA) and asymmetric dimethylarginine (aDMA).

Cofactor-associated arginine methyltransferase 1 (CARM1) is a member of the family of protein arginine *N*-methyltransferases (PRMTs), responsible for the methylation of arginine residues in a variety of nuclear protein substrates, including histone tails, RNA binding proteins, and splicing factors.^1,2^ Arginine methylation in histones and other nuclear proteins plays an important role in regulating a range of cellular processes, including gene regulation, signal transduction, RNA processing, and DNA repair.^3,4^ PRMTs can be classified into three types based on their primary product formation: type I PRMTs result in both ω-*N*^*G*^-monomethyl arginine (MMA) and asymmetrically ω-*N*^*G*^,*N*^*G*^ dimethylated arginine (aDMA), type II PRMTs catalyze the formation of MMA and symmetrical ω-*N*^*G*^,*N′*^*G*^-dimethylarginine (sDMA), and type III PRMTs exclusively form MMA.^5,6^ As a type I PRMT, CARM1 catalyzes the transfer of the methyl group from *S*-adenosyl-l-methionine (AdoMet) to first generate MMA followed directly by a second methylation step, resulting in the formation of aDMA (Figure 1). The methyl group transfer from AdoMet to the protein substrate generates the byproduct *S*-adenosyl-l-homocysteine (AdoHcy), which in turn can inhibit CARM1 as a feedback inhibitor.^7^

The aberrant expression of CARM1 has been linked to a variety of disease states, most prominently in the field of cancer. CARM1 overexpression is linked to ovarian, colorectal, prostate, and lung cancers.^8−10^ In addition, CARM1 was found to promote cell proliferation of ERα-positive breast cancer cells.^11^ These findings have led to interest in CARM1 as a potential therapeutic target for the treatment of cancer. To facilitate the development of inhibitors of CARM1, reliable, specific, and rapid analytical methods for characterizing its activity are vital.

Generally, analytical methods for the detection of methyltransferase activity focus on the detection of enzymatic byproduct AdoHcy. Several high-throughput assays are available for the detection of AdoHcy, either directly by chromatographic means,^12^ or indirectly, using enzyme-coupled assays in which AdoHcy formation leads to a luminescent or fluorescent signal.^13,14^ Using such an approach, we recently investigated the use of a commercially available assay kit (MTase Glo) for the purposes of studying CARM1 activity but were not able to achieve consistent results (data not shown). We attribute this to the previously noted high background signal encountered with this method owing to the auto-methylating ability of CARM1 at its own arginine residue R551.^15^ These findings suggested to us that methods relying on the detection of AdoHcy formation are not optimal for the quantification of CARM1 activity. For this reason, we were inspired to develop an alternative assay focused on the direct detection of the methylated products formed by CARM1.

Substrate methylation can be quantified using existing methods, for example, through the use of radiolabeled ^3^H-AdoMet^16^ to measure direct methyl group addition or indirectly through the use of antibodies developed against specific methylated epitopes.^17^ There are, however, several disadvantages to these assays. While compatible with high-throughput screening (HTS), radiometric approaches require strict operating conditions, radio-protected equipment, and specific laboratory setups. In comparison, while antibody-based ELISA assays avoid the use of radioactivity, they are expensive and involve complex experimental protocols that are not suitable for high-throughput screening. To address these shortcomings, we here describe the development of a rapid, straightforward, and sensitive CARM1-specific assay. Specifically, our method relies upon the direct detection of the dimethylated products formed when substrate peptides derived from poly(A)-binding protein 1 (PABP1) are incubated with CARM1 and AdoMet. Using an LC-MS-based approach, the enzymatic products are readily detected via multiple reaction monitoring (MRM) and quantified by comparison to a hexadeuteromethylated species serving as internal standard. MRM is a technique widely used in quantitative proteomics because of its high selectivity using two levels of mass detection, high sensitivity, and wide dynamic range.^18^ We further demonstrate the suitability of this rapid and direct analytical method in characterizing CARM1 inhibition by evaluating a number of established CARM1 inhibitors. Notably, the results obtained with our assay were found to compare well with those obtained when using a more operationally complex antibody-based chemiluminescent method. The analytical method here reported provides high selectivity and sensitivity in the characterization of CARM1 activity and offers a simplified approach to screening for inhibitors of CARM1.

## Experimental Section

### CARM1 Cloning, Expression, and Purification

The *Mus musculus* CARM1 (mmCARM1) gene sequence corresponding to the PRMT core (residues 130–497, mmCARM1_130–497_) were amplified by PCR from the original GST-CARM1 construct.^19^ The sequences were cloned in the pDONR207 (Invitrogen) vector using a BP reaction (Gateway Cloning, Life Technologies). The positive clones were confirmed by sequencing (GATC). The sequences were subcloned in a pDEST20 vector using an LR reaction. The resulting recombinant protein is harboring an amino-terminal glutathione S-transferase (GST) tag followed by a Tobacco etch virus (TEV) protease cleavage site. DH10Bac competent cells containing the baculovirus genome were transformed with the pDEST20-CARM1 plasmids and plated onto LB agar media containing 15 mg·mL^–1^ tetracycline, 7 mg·mL^–1^ gentamicin, 50 mg·mL^–1^ kanamycin, 25 mg·mL^–1^ X-Gal, and 40 mg·mL^–1^ IPTG. Bacmid DNA purified from recombination-positive white colonies was transfected into Sf9 cells using the Lipofectin reagent (Invitrogen). Viruses were harvested 10 days after transfection. Sf9 cells were grown at 300 K in suspension culture in Grace medium (Gibco) using Bellco spinner flasks. sf9 cell culture (1 L, 0.8 × 10^6^ cells·mL^–1^) was infected with recombinant GST-mmCARM1 virus with an infection multiplicity of 1. The cells were harvested 48 h post-infection. Cell lysis was performed by sonication in 50 mL of buffer *A* [50 mM Tris-HCl pH 8.0, 250 mM NaCl, 5% glycerol, 5 mM TCEP, 0.01% NP40 and anti-proteases (Roche, Complete, EDTA-free)], and cellular debris was sedimented by centrifugation of the lysate at 40 000*g* for 30 min. The supernatant was incubated overnight at 277 K with 2 mL of glutathione Sepharose resin (GE Healthcare). After a short centrifugation, the supernatants were discarded, and the beads were poured in an Econo-column (Bio-Rad). After two washing steps with 10 mL of buffer A, 2 mL of buffer *A* supplemented with in-house produced TEV protease was applied to the columns and digestion was performed for 4 h at 303 K with gentle mixing. The digest was concentrated with an Amicon Ultra 10K (Milipore), loaded on a gel-filtration column (HiLoad 16/60 Superdex S200, GE Healthcare), and eluted at 1 mL·min^–1^ with buffer B [20 mM Tris-HCl pH 8.0, 100 mM NaCl, 1 mM TCEP] using an ÄKTA Purifier device (GE Healthcare). Fractions containing mmCARM1_130–497_ were pooled and concentrated to 7.75 mg·mL^–1^.

### Peptide Synthesis

**Figure 2:**
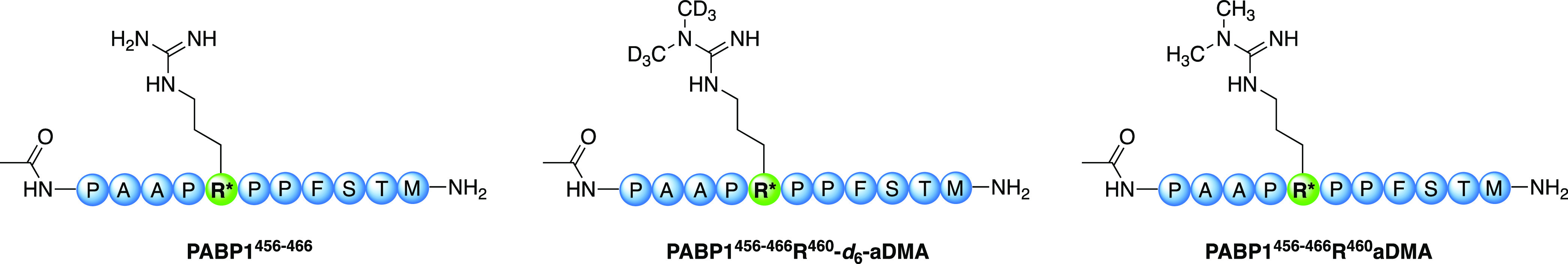
Structures of the PABP1^456–466^ substrate, the PABP1^456–466^-R^460^-*d*_*6*_-aDMA internal standard, and the PABP1^456–466^-R^460^-aDMA reference standard.

The PABP1^456–466^ peptides (Figure 2) used in the study were prepared via solid-phase peptide synthesis (SPPS) using a CEM Liberty Blue microwave-assisted peptide synthesizer. The Fmoc-protected Rink amide AM resin (0.1 mmol) was first swollen in 10 mL of a 1:1 mixture of DMF/DCM for 5 min, drained, and treated with 20 vol % piperidine (10 mL) in DMF for 65 s at 90 °C, drained, and washed with DMF (3 × 5 mL). The resin was then treated with a solution of Fmoc-Met-OH (0.2 M, 2.5 mL, 5 equiv), DIC (1 M, 1 mL, 10 equiv), and Oxyma (1 M, 0.5 mL, 5 equiv) in DMF (4 mL) at 76 °C for 15 s before the temperature was increased to 90 °C for an additional 110 s before being drained. To achieve maximal yield, each amino acid was double-coupled according to the previous cycle. Following Fmoc removal with 20 vol % piperidine (10 mL) in DMF for 65 s at 90 °C, the resin was drained and washed with DMF (3 × 5 mL) after which the subsequent amino acids were coupled. All Fmoc amino acids were obtained commercially with the exception of Fmoc-*d*_6_-aDMA(Pbf)-OH, which was prepared as described in the Supporting Information. After coupling and deprotection of the final amino acid, the N-terminus was acetylated on resin using acetic anhydride (0.5 mL) and DiPEA (0.85 mL) in DMF (10 mL) for 120 s at 65 °C. Then, the resin was washed three times with DMF (10 mL). The final peptides were cleaved from the resin using a mixture of TFA/water/TIPS (95:2.5:2.5) under shaking for 2 h at room temperature. The resin was filtered over cotton and washed with TFA (2 × 0.5 mL). The crude peptides were precipitated in a mixture of MTBE/hexane (1:1) and pelleted by centrifugation (5 min at 4500 rpm). The pellet was then washed twice with MTBE/hexane (1:1) (50 mL), centrifuged (5 min at 4500 rpm), and dried under a nitrogen flow. The crude peptides were purified by prep-HPLC and characterized by LC-MS and HRMS. The final yield of the peptides ranges from 30 to 40%.

### Enzymatic Activity Assay

Enzyme activity assays were performed with CARM1 (286 nM corresponding to 11.68 ng/μL) in 20 mM Tris buffer (pH 8) containing 50 mM Tris NaCl, 1 mM EDTA, 3 mM MgCl_2_, 0.1 mg/mL BSA, and 1 mM dithiothreitol (DTT). The enzyme mixture (20 μL) was added to the substrate mixture (20 μL) containing the PABP1^456–466^ substrate peptide and AdoMet (final concentrations of 12 and 10 μM, respectively) followed by incubation for 2 h at room temperature. The reaction was subsequently quenched by the addition of 30 μL of the reaction mixture to 10 μL of a 0.1% formic acid solution (pH 2). After the addition of the deuterated internal standard in water (100 nM, 40 μL) and mixing for 2 min, the samples were centrifuged for 5 min at 3000 rpm. The supernatant (60 μL) was transferred to a new 96-well plate and analyzed.

### LC-MS Method for the Analysis of Methylated Peptides

LC-MS analysis was performed on a Shimadzu LC-20AD system with a Shimadzu Shim-Pack GIST C18 column (3.0 × 150 mm^2^, 3 μm particle size) at 30 °C connected to a Shimadzu 8040 triple quadrupole mass spectrometer with an electrospray ionization (ESI) source. The products were eluted with a water–acetonitrile gradient moving from 20 to 92% acetonitrile (0.1% FA) over 6 min at a flow rate of 0.5 mL·min^–1^. The injection volume was 10 μL. The ionization source was operated in positive mode using an interface voltage of 4.5 kV, nebulizing gas at 1.5 L/min, drying gas at 15 L/min, and a desolvation line (DL) temperature of 250 °C. The MRM parameter optimization was performed using both the analyte (PABP1^456–466^-R^460^-aDMA) and hexadeuterated internal standard (PABP1^456–466^-R^460^-*d*_6_-aDMA). The results of this optimization, which include precursor ion scanning, collision energy, and Q1 and Q3 scanning, are summarized in Table 1.

**Table 1:** Optimized MRM Parameters for the PABP1 Analyte and Internal Standard^a^

compounds		Q1 (*m*/*z*)	Q3 (*m*/*z*)	Q1 PreBias (V)	CE (V)	Q1 PreBias (V)
PABP1^456−466^-R^460^-aDMA	analyte	620.85	211.00	–28	–29	–22
			140.00	–28	–47	–25
			282.00	–28	–20	–30
PABP1^456−466^-R^460^-*d*_6_-aDMA	standard	623.75	210.95	–28	–28	–22
			140.00	–24	–45	–26
			282.00	–28	–19	–29

a The interface voltage was set at 4.5 kV for all of the compounds; dwell time was 100 ms. Q1: quadrupole 1, Q3: quadrupole 3, *m*: mass, *z*: charge, CE: collision energy.

### Analytical Method Validation (Linearity, Limit of Detection, Accuracy, and Precision)

Analysis of the PABP1^456–466^-R^460^-aDMA peptide was validated between 16 and 512 nM for within and between run accuracy and precision, the linearity of the calibration curve, the sample recovery, and the limit of detection. Linearity was performed with calibration points consisting of 1, 2, 4, 8, 16, 32, 64, 128, 256, 512, and 1024 nM PABP1^456–466^-R^460^-aDMA peptide dissolved in water. Samples for analysis were worked up as described above (see Enzymatic Activity Assay section) and analyzed with the LC-MS/MS method. Area ratios of PABP1^456–466^-R^460^-aDMA and the hexadeuterated internal standard were assessed and plotted versus concentration. Linearity was assessed visually and by calculation of the coefficient of determination *R*^2^, which should be >0.98. The limit of detection (LOD) was determined by the samples corresponding to a signal-to-noise ratio (S/N) of 3.

Quality control (QC) samples consist of PABP1^456–466^-R^460^-aDMA concentrations of 16, 64, and 512 nM and enzymatic reaction buffer (20 mM Tris buffer pH 8, 50 mM NaCl, 1 mM EDTA, 3 mM MgCl_2_, 0.1 mg/mL BSA and 1 mM DTT). QC samples for analysis were worked up as described above in the enzymatic reaction assay section and analyzed with the LC-MS/MS method. To evaluate the precision and accuracy of the quantification of PABP1^456–466^-R^460^-aDMA, concentration values were recalculated for QC using calibration curves. Intrarun accuracy and precision tests were performed using PABP1^456–466^-R^460^-aDMA concentrations of 16, 64, and 512 nM. Accuracy and precision tests were performed in sixfold per concentration in one run and in onefold per concentration in three separate runs. The acceptance criteria of the accuracy results were 85–115%, and of the precision results <15%. The limit of detection was calculated to be 1.55 nM, and the method was linear between 8 and 512 nM with an *R*^2^ of 0.996 (Table 2). The lowest concentration giving a reliable and accurate signal was found to be 16 nM.

**Table 2:** Validation Parameters of the MRM Method for the Detection of PABP1^456–466^-R^460^-aDMA

[QC] (nM)	*R*^2^	
8–512	0.996	

	accuracy (%)	precision CV (%)
within run (*n* = 6)		
16	113.7	2.1
64	87.0	4.7
512	94.4	5.6
between runs (*n* = 3)		
16	106.2	0.1
64	97.5	2.3
512	91.2	4.9
limit of detection (S/N ≥ 3)	1.55 nM	

### Enzyme Inhibition Assay

The CARM1 inhibition assays were performed using a number of established, commercially available CARM1 inhibitors as well as a series of peptidomimetic inhibitors recently reported by our group.^20^ When using the assay to characterize CARM1 inhibition, the substrates were set at concentrations near their calculated *K*_M_ values (12 μM for the PABP1^456–466^ peptide and 10 μM for AdoMet). The inhibitors were tested at 10 different concentrations that were selected based on their published IC_50_ values. For commercially available inhibitors that were not soluble in water, stock solutions were prepared in DMSO and diluted to a final DMSO concentration of <1% in the assay mixture. CARM1 (20 μL) and inhibitors (10 μL) were incubated for 15 min at room temperature, followed by the addition of a mixture of peptide substrate and AdoMet (10 μL) to start the reaction. The mixture was incubated for 2 h at room temperature, and the reaction was subsequently quenched by the addition of 30 μL of the reaction mixture to 10 μL of a 0.1% formic acid solution (pH 2). After the addition of the deuterated internal standard in water (100 nM, 40 μL) and mixing for 2 min, the samples were centrifuged for 5 min at 3000 rpm. The supernatant (60 μL) was transferred to a new 96-well plate and analyzed by LC-MS as described above. Negative controls (no enzyme) and positive controls (no inhibitor) were included in each plate.

### Data Analysis

The data obtained from the MRM method included a linearity line with 10 different concentrations of reference standard (from 8 to 512 nM) and a fixed concentration of internal standard (100 nM). These data points were subjected to weighted regression (1/*x*^2^). The intercept and slope were used for the determination of the measured concentrations.

For quantification of the methylated product, the area ratio of analyte to internal standard was calculated and quantified using the linearity line obtained with the reference standards. The concentrations were then converted to enzyme velocity in nmole produced/h/mg CARM1 using eq 1 with the concentration of the methylated product in nM, time in minutes, and enzyme concentration in mg/L.
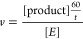
1Calculation of *V*_max_ and *K*_m_ was done using GraphPad Prism 6 following nonlinear (Michaelis–Menten) regression analyses using eq 2.
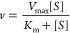
2*k*_cat_ was calculated from *V*_max_ using eq 3, with *V*_max_ in nmol/h/mg enzyme and enzyme concentration in mg/L. To obtain *k*_cat_ with units of s^–1^, the maximal velocity (*V*_max_) is divided by 3600.
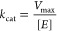
3The percentage inhibition was plotted as a function of inhibitor concentration and fit using a nonlinear regression analysis of the sigmoidal dose–response curve generated using the normalized data and a variable slope following eq 4.
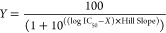
4where *Y* is the percent inhibition, *X* is the logarithmic concentration of the inhibitors, and Hill Slope is the slope factor or Hill coefficient. The IC_50_ value was determined by the concentration resulting in half-maximal percent activity. Values reported include the standard errors of the mean (S.E.M., calculated using the symmetrical CI function in GraphPad Prism 6) indicating the precision of the mean values obtained.

## Results and Discussion

### Analytical Method Development

To achieve a rapid and direct analytical method for the quantification of CARM1 activity, we have developed an LC-MS method using multiple reaction monitoring (MRM) analysis and optimized it to obtain maximal detection sensitivity and accuracy. In search of a peptide substrate suitable for use in an LC-MS-based activity assay for CARM1, we initially focused our attention on peptides derived from histone H3. Tail peptides from H3 are well-characterized substrates of CARM1, with preferential methylation occurring at arginine residue H3-R^17^.^19,21−23^ To assess the suitability of H3 peptides with the envisioned LC-MS detection method, we first synthesized H3^16–30^ incorporating an asymmetrically dimethylated arginine residue at arginine 17. Subsequent analysis of the H3^16–30^-R^17^aDMA peptide by LC-MS was found to produce a distribution of *m*/*z* values rather than a major single precursor ion owing to the presence of other arginine and lysine residues in the sequence. This in turn led to a significant reduction in signal as even when selecting for the major precursor ion, ∼75% of total signal was lost. No significant improvement was observed when using buffers at different pH in an attempt to tune the charge distribution of the peptide (data not shown). We therefore opted to evaluate different substrate peptides not based on H3 but rather derived from poly(A)-binding protein 1 (PABP1), a protein known to be efficiently methylated by CARM1 at arginine residues 455 and 460.^24,25^ Notably, the PABP1 sequences PABP1^447–459^ and PABP1^456–466^ do not include any additional positively charged residues other than the arginine residues R^455^ and R^460^, respectively. To this end, PAPB1^447–459^-R^455^aDMA and PAPB1^456–466^-R^460^aDMA were synthesized and analyzed by LC-MS. Based on peak shape and signal intensity, PAPB1^456–466^-R^460^aDMA was identified as the preferred analyte and used for optimization. In contrast to the histone H3 sequence, mass analysis of this PABP1 sequence yielded a single major peak (*m*/*z* = 620.850, corresponding to [M + 2H]^2+^), which was subsequently selected as the precursor ion for further MRM optimization (Figures S2 and S3).

During the optimization of the MRM method, we examined the influence of mobile phase composition and pH, column temperature, and flow rate on the elution profile of the PAPB1^456–466^-R^460^aDMA standard. Initial attempts employed isocratic elution with a mobile phase consisting of 25% acetonitrile containing 20 mM NH_4_Ac (pH 7, flow rate is 0.5 mL·min^–1^ at 30 °C) and 20 mM NH_4_Ac (pH 9, flow rate is 0.5 mL·min^–1^ at 30 °C), respectively. These conditions yielded a broad saw-like peak for the peptidic analyte. When the mobile phase was changed to 25% acetonitrile containing 0.1% formic acid (pH 2), a smooth peak resulted but still gave a broad signal with significant peak tailing. To improve peak shape, we subsequently evaluated gradients of acetonitrile in aqueous formic acid (0.1%). This led to an optimized method employing a gradient moving from 20 to 92% acetonitrile in aqueous formic acid (0.1%) (pH 2), which reliably gave a sharp and symmetrical peak for PAPB1^456–466^-R^460^aDMA. Variation of the slope of the gradient (between 6 and 20 min) did not significantly affect the peak shape, allowing for a convenient run time of 6 min. Subsequently, we examined the column temperature (up to 60 °C) and flow rate (0.5 and 1 mL·min^–1^), but this provided no significant improvement. The final conditions were therefore set on a method with a run time of 6 min and a gradient of 20–92% acetonitrile in water containing 0.1% formic acid with a flow rate of 0.5 mL·min^–1^ at 30 °C. The MRM parameters generated for the PAPB1^456–466^-R^460^aDMA through an automated methodology of the mass spectrometer were incorporated in the LC-MS method.

### Internal Standards

As an internal standard, we prepared the hexadeuterated form of the analyte, PAPB1^456–466^-R^460^-*d*_6_-aDMA. In doing so, any changes in the analyte signal resulting from variation in the workup or the analytical method (e.g., due to matrix effects, ion suppression, precipitation, or nonspecific binding) can be corrected. Isotopically labeled compounds have the same chromatographic behavior and show the same ionization and fragmentation pattern as their nonlabeled counterparts but can be separated based on their mass difference. The synthesis of PAPB1^456–466^-R^460^-*d*_6_-aDMA was conducted for the nondeuterated species with the exception that a hexadeuterated aDMA building block was required, which was prepared following protocols previously reported by our group.^26,27^

### Optimization of the Enzymatic Activity Assay

The conditions of the enzymatic activity assay were optimized with respect to buffer composition, reaction time, and workup. The optimized buffer consists of 20 mM Tris buffer (pH 8) containing 50 mM NaCl, 1 mM EDTA, 3 mM MgCl_2_, 0.1 mg/mL BSA, and 1 mM DTT. The addition of DTT was vital for avoiding disulfide bond formation, and the addition of BSA was found necessary to keep CARM1 in its active form by blocking aggregation and reducing unspecific binding of the CARM1 to the well plate. Sample workup consisted of quenching the enzyme reaction by the addition of 0.1% formic acid solution (known to be compatible with the MS conditions of the assay^28^) and addition of the internal standard.

To maximize the signal for the enzymatic reaction, a screen was performed to establish both the optimal concentration of enzyme and incubation time. For CARM1, the half-maximal effective concentration (EC_50_) determination was performed using CARM1 enzyme at concentrations of 21, 43, 86, 172, 344, 688, 1376, and 2752 nM. Substrates were fixed at 100 μM PAPB1^456–466^ and 10 μM AdoMet, and samples were taken every 15 min for 2 h. The CARM1 EC_50_ value was thus established to be 286.2 ± 8.1 nM corresponding to 11.7 ± 0.33 ng/μL (Figure S3), which is in good agreement with the final concentration of CARM1 used in the commercially available chemiluminescent assay kit (BPS Bioscience, Catalog #52041L; CARM1 concentration is 10 ng/μL corresponding to 245 nM). Notably, the sigmoidal shape of the curve obtained for the activity of CARM1 may be attributed to enzyme dimerization, for which a minimal enzyme concentration could be necessary. The necessity of PRMT dimerization in achieving full activity has been demonstrated for PRMT1 and may also be necessary for the activity of CARM1.^29−31^ For the determination of the *K*_M_ value of PABP1^456–466^ the formation of the methylated product was analyzed in the presence of a fixed concentration of 100 μM AdoMet and PABP1^456–466^ applied over a concentration range of 0.05–100 μM. For the determination of the *K*_M_ value of AdoMet, the methylated substrate was analyzed in the presence of a fixed concentration of 100 μM PABP1 and AdoMet concentrations ranging from 0.05 μM to 100 μM. The *K*_M_^app^ values thus obtained were 12.03 ± 2.28 μM for PABP1^456–466^ and 5.46 ± 0.01 μM for AdoMet (Figure S3). Based on these findings, when performing the subsequent inhibition studies, CARM1 was used at a concentration of 286 nM, while the substrate concentrations were fixed at 12 μM PABP1 and 10 μM AdoMet.

### Inhibitor Studies

**Figure 3:**
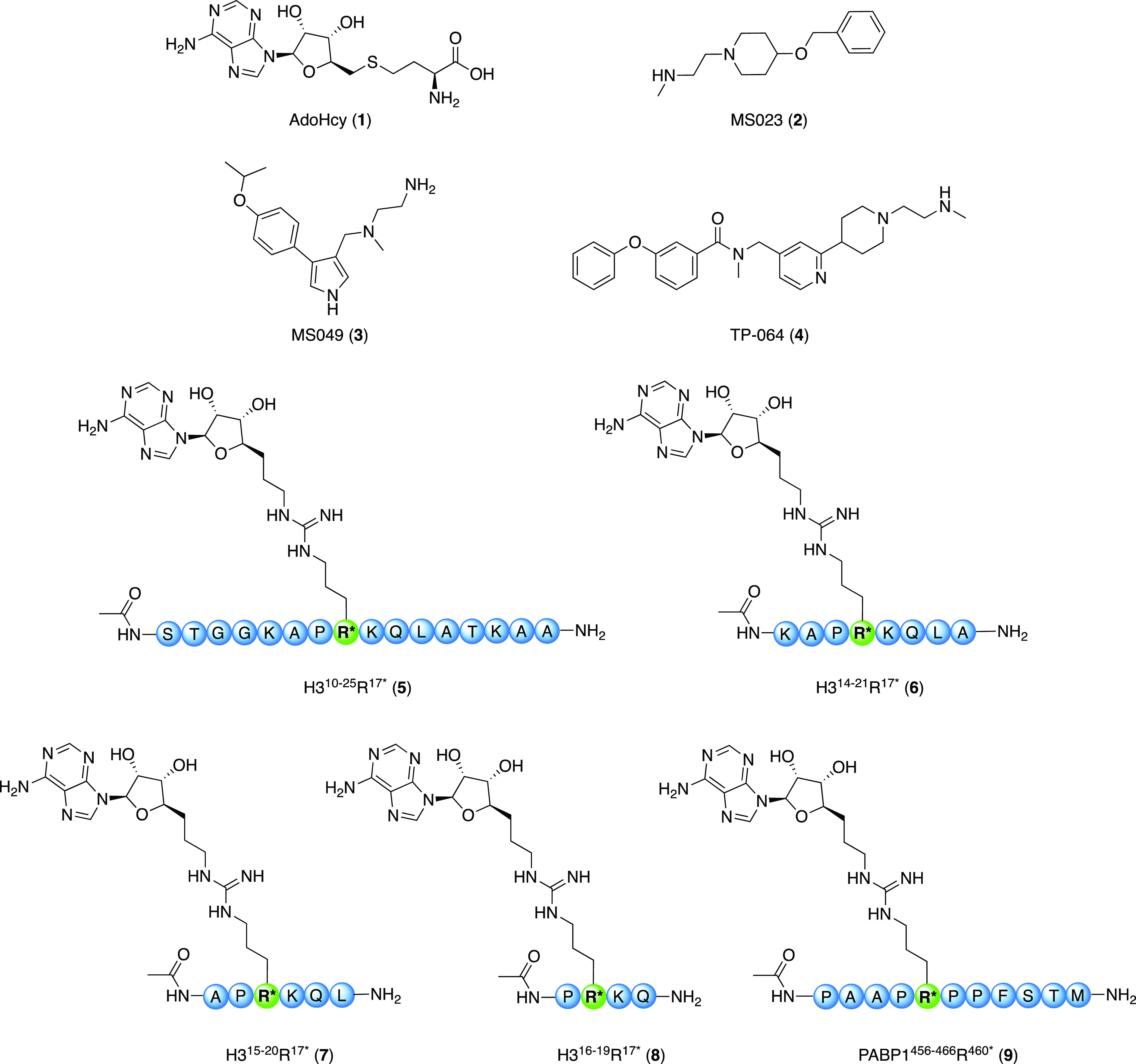
Overview of the chemical structures of reported small-molecule CARM1 inhibitors AdoHcy (**1**), MS023 (**2**), MS049 (**3**), and TP-064 (**4**) and peptidomimetic inhibitors H3^10–25^R^17*^ (**5**), H3^14–21^R^17*^ (**6**), H3^15–20^R^17*^ (**7**), H3^16–19^R^17*^ (**8**), and PABP1^456–466^R^460*^ (**9**).

We next applied the assay in assessing the inhibition of CARM1 by a number of known inhibitors of varying potencies including AdoHcy (**1**), MS023 (**2**), MS049 (**3**), TP064 (**4**), and a series of recently reported peptidomimetic CARM1 inhibitors (**5–9**) (Figure 3).^20,32−35^ For the purpose of generating IC_50_ curves for these inhibitors, concentration ranges were set according to previously reported IC_50_ values. The inhibitors were first incubated with CARM1 for 15 min at room temperature before the enzyme reaction was initiated by the addition of the AdoMet/PABP1^456–466^ substrate mixture. On the basis of the residual CARM1 activity measured, inhibition curves were generated and the IC_50_ values were determined (Table 3). To evaluate the suitability of the method for the determination of CARM1 inhibition, the IC_50_ values were compared with those obtained using a commercially available chemiluminescent ELISA kit. The conditions used with this kit are comparable to those used here in terms of enzyme concentrations, but a slightly lower AdoMet concentration is applied in the kit (1 μM versus 10 μM). In addition, the peptide substrate and detection method employed in the ELISA kit are inherently different. The kit employs a histone H3-derived peptide that is covalently linked to the bottom of the well plate, and as such, no substrate concentration is given. Product formation in turn is detected using specific antibodies that recognize aDMA formation at arginine residue H3-R^17^.

**Table 3:** Inhibition Data for Compounds **1–9** against CARM1 Tested by the MRM LC-MS Assay and the Antibody-Based ELISA Assay

inhibitor		CARM1 IC_50_ values (μM)^a^	
number	compound name	MRM LC-MS assay	ELISA assay
1	AdoHcy	0.873 ± 0.339	0.276 ± 0.074 (ref (27))
2	MS023	0.327 ± 0.034	0.101 ± 0.011 (this work)
3	MS049	0.082 ± 0.008	0.028 ± 0.003 (this work)
4	TP064	0.162 ± 0.010	0.037 ± 0.008 (this work)
5	H3^10–25^R^17*^	0.770 ± 0.098	0.290 ± 0.021 (ref (20))
6	H3^14–21^R^17*^	0.403 ± 0.028	0.287 ± 0.048 (ref (20))
7	H3^15–20^R^17*^	0.617 ± 0.057	0.143 ± 0.020 (ref (20))
8	H3^16–19^R^17*^	1.456 ± 0.021	0.346 ± 0.044 (ref (20))
9	PABP1^456–466^-R^460*^	0.212 ± 0.025	0.090 ± 0.016 (ref (27))

a IC_50_ values reported in μM from duplicate data obtained from a minimum of seven different concentrations ± standard deviation (SD). Full inhibition curves are provided in the Supporting Information.

The results of the inhibitor screen are summarized in Table 3 and show that the potency trend for the IC_50_ values obtained with the MRM LC-MS assay corresponds very well with that obtained with the ELISA-based method (Table 3). The absolute IC_50_ values measured via the MRM LC-MS assay were found to be generally 2–4 times higher than those obtained via the ELISA assay, an effect we ascribe to the differences in assay conditions and methodology. The most notable differences between the MRM LC-MS and ELISA assays lie in the AdoMet concentrations and peptide substrates used. The MRM LC-MS method here reported uses a 10-fold higher concentration of AdoMet, which likely impacts the IC_50_ values measured. Furthermore, MRM LC-MS assay detects the CARM1 catalyzed methylation of a PAPB1-derived substrate while the ELISA method employs an H3-based peptide substrate. Notably, the published *K*_M_ value of CARM1 for such H3 substrates (112 μM)^36^ is 10-fold higher than that of PABP1-based substrates (*K*_M_ = 12 μM, this work). In the context of inhibition assays, the higher affinity of CARM1 for PABP1-based substrates versus those derived from H3 is also likely to impact the relative IC_50_ values measured for competitive inhibitors.

## Conclusions

We here describe the development of a direct, specific, and convenient analytical method for measuring the activity of CARM1. The LC-MS-based method applies multiple reaction monitoring (MRM) for the detection and quantification of a methylated peptide substrate (PAPB1^456–466^-R^460^aDMA). The assay presents a significant simplification over existing ELISA and radiometric methods while benefitting from high sensitivity and convenient sample preparation. Compared with the widely used radiolabeled AdoMet assay, the MRM LC-MS assay is not restricted by specialized operational and laboratory conditions. We have also demonstrated the application of the MRM LC-MS method in assaying the inhibitory activity of a selection of known CARM1 inhibitors by generating CARM1 inhibition curves. The IC_50_ values obtained were found to be comparable with published values and with values obtained with the commercially available ELISA kit. Considering the growing body of evidence for CARM1 as a therapeutic target, the MRM LC-MS assay here described represents a valuable addition to the tools available for the identification of CARM1 inhibitors. Furthermore, the 6-min run time of the MRM LC-MS assay allows for the convenient assessment of focused libraries numbering in the tens to hundreds of compounds. While HTS campaigns for CARM1 inhibitor identification typically rely on alternative methods such as radiometric detection, the CARM1 specificity of the MRM LC-MS assay makes it very well suited for hit validation purposes. In addition, the approach here described should be widely applicable in the development of assays for other methyltransferases provided that compatible substrates are available.

## Supporting Information Available

Building block synthesis (Scheme S1); analytical HPLC (Figures S1 and S2) and HRMS of characterization of peptides and NMR characterization of the Fmoc-*d*_6_-aDMA(Pbf)-OH building block; kinetic analysis of CARM1 substrates (Figure S3); and IC_50_ traces of all of the compounds (Figure S4) (PDF)

## Accession Codes

CARM1: UniProt ID Q86X55

## Abbreviations

CARM1: coactivator-associated arginine methyltransferase 1
MMA: ω-*N*^*G*^-monomethyl arginine
aDMA: asymmetrically ω-*N*^*G*^,*N*^*G*^ dimethylated arginine
sDMA: ω-*N*^*G*^,*N′*^*G*^ −dimethylarginine
PABP1: poly(A)-binding protein 1
MRM: multiple reaction monitoring
LC-MS: liquid chromatography mass spectrometry
PRMT: protein arginine methyltransferase
RP-HPLC: reversed-phase high-performance liquid chromatography
AdoHcy: *S*-adenosyl-l-homocysteine
AdoMet: *S*-adenosyl-l-methionine
SPPS: solid-phase peptide synthesis
TFA: trifluoroacetic acid
TIPS: triisopropylsilane
DMF: dimethylformamide
DCM: dichloromethane
EDTA: ethylenediaminetetraacetic acid
DTT: dithiothreitol
BSA: bovine serum albumin
ELISA: enzyme-linked immunosorbent assay

## Notes

Samples of the PABP1^456–466^ substrate peptide, PABP1^456–466^-R^460^-*d*_6_-aDMA internal standard, and PABP1^456–466^-R^460^-aDMA reference standard are available upon request.
