# Supplementary material for: A Direct Assay for Measuring the Activity and Inhibition of Coactivator-Associated Arginine Methyltransferase 1
Source: Biochemistry. 2022 May 17;61(11):1055–63. doi: 10.1021/acs.biochem.2c00075 (PMC9178793; doi:10.1021/acs.biochem.2c00075)
Supplement: Supplementary file 1 — bi2c00075_si_001.pdf [file bi2c00075_si_001.pdf]

**Supporting Information for:**

# A Direct Assay for Measuring Activity and Inhibition of Coactivator Associated Arginine Methyltransferase 1

*Yurui Zhang,[1,‡] Matthijs J. van Haren,[1,‡] Nils Marechal,[2] Nathalie Troffer-Charlier,[2] Vincent Cura,[2] Jean Cavarelli[2] and Nathaniel I. Martin[1,*]*

*[1]Biological Chemistry Group, Institute of Biology Leiden, Leiden University, Sylviusweg 72, 2333 BE Leiden, The Netherlands *E-mail: n.i.martin@biology.leidenuniv.nl*

*[2]Department of Integrated Structural Biology, Institut de Génétique et de Biologie Moléculaire et Cellulaire, Université de Strasbourg, CNRS UMR 7104, INSERM U 1258, Illkirch, F-67404, France*

*[‡]These authors contributed equally to this work*

## Table of Contents

Chemical synthesis ........ S2

LC-MS traces of analyte and internal standard ........ S4

Kinetic analysis of CARM1 substrates ........ S5

HPLC and High Resolution Mass Spectrometry data for PABP1 peptides ........ S6

$IC_{50}$ curves for compounds **1-9** ........ S8

NMR Data of Fmoc-$d_6$-aDMA-OH ........ S10

References ........ S11

# Chemical synthesis

## Building block Synthesis

The Fmoc-$d_6$-aDMA(Pbf)-OH building block was synthesized from commercially available Fmoc-Orn(Boc)-OH **S1** following the synthetic route for Fmoc-aDMA(Pbf)-OH as previously described (**Scheme S1**).[1] Briefly, compound **S1** was transformed into allyl ester **S2** by treatment with allyl alcohol, HOBt, DMAP, and DCC in THF. Subsequently, allyl ester **S2** was treated with TFA/DCM (2:1) to remove the Boc group and reacted with 2,2,4,6,7-pentamethyldihydrobenzofuran-5-sulfonyl isothiocyanate (Pbf-NCS) to form Pbf-protected thiourea **S3**. Finally, compound **S3** was reacted with dimethyl-$d_6$-amine hydrochloride in the presence of 1-ethyl-3-(3-dimethylaminopropyl)-carbodiimide (EDCI) to form the intermediate guanidine species which was treated directly with tetrakis(triphenylphosphine) palladium(0) to form Pbf-protected Fmoc building block **S4** which was used for solid phase peptide synthesis.

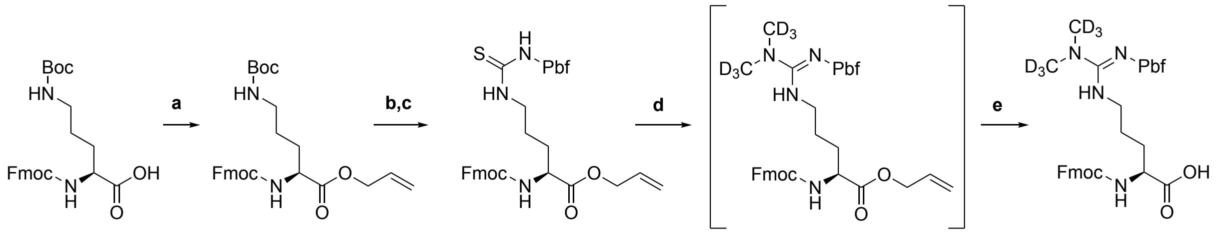


S1 S2 S3 S4

**Scheme S1.** Synthesis of Fmoc-$d_6$-aDMA. **a**. allyl alcohol, HOBt, DMAP, DCC, THF, overnight, (yield 62%); **b**. TFA/DCM(2:1),1h; **c**. Pbf-NCS in DCM (0.1 M), 2h, (yield 60%); **d**. EDCI, bis(methyl-$d_3$)amine hydrochloride, DCM, overnight; **e**. $Pd(PPh_3)_4$, *N*-methylaniline, $N_2$, overnight, (yield 85% over 2 steps).

### (E)-N2-(((9H-fluoren-9-yl)methoxy)carbonyl)-N$^{\omega}$,N$^{\omega}$-bis(methyl-d3)-N$^{\omega'}$-((2,2,4,6,7-pentamethyl-2,3-dihydrobenzofuran-5-yl)sulfonyl)arginine (S4)

To a solution of compound **S3** (610 mg, 0.86 mmol) in DCM (30 mL), 1-ethyl-3-(3-dimethylaminopropyl)carbodiimide (269 mg, 1.73 mmol) and bis(methyl-$d_3$)amine hydrochloride (151 mg, 1.73 mmol) were added. The mixture was stirred overnight at room temperature. The mixture was

diluted with DCM (50 mL), washed with 10% citric acid (2 x 20 mL) and saturated sodium bicarbonate (2 x 20 mL) and dried over sodium sulfate. The organic solvent was removed and the residue was redissolved in THF (40 mL). The mixture was treated with *N*-methylaniline (238 μL, 2.31 mmol), followed by addition of tetrakis(triphenylphosphine) palladium(0) (41.4 mg, 0.04 mmol). The mixture was protected from light and stirred under nitrogen at room temperature. After TLC indicated completion of the allyl ester removal, the solvent was removed under reduced pressure and purified by column chromatography (2% methanol in DCM) to yield compound **S4** (500 mg, 85%) as a white foam. HRMS (m/z): $[M+H]^+$ calculated for $C_{36}H_{39}D_6N_4O_7S^+$, 683.3385, found 683.3345. $^1$H NMR (500 MHz, $CDCl_3$) δ 8.17 (s, 1H), 7.64 (d, J = 7.6 Hz, 2H), 7.48 (dd, J = 7.6, 4.5 Hz, 2H), 7.28 (m, J = 7.3 Hz, 2H), 7.19 – 7.15 (m, 2H), 6.00 (d, J = 7.7 Hz, 1H), 4.22 (m, J = 7.1, 3.1 Hz, 2H), 4.15 (d, J = 6.4 Hz, 1H), 4.08 (t, J = 7.3 Hz, 1H), 3.30 – 3.07 (m, 2H), 2.84 (s, 2H), 2.38 (s, 3H), 2.32 (s, 3H), 1.98 (s, 3H), 1.81 – 1.48 (m, 4H), 1.35 (s, 6H). $^{13}$C NMR (126 MHz, $CDCl_3$) δ 174.33, 161.58, 161.28, 160.98, 160.79, 160.67, 156.89, 155.62, 143.84, 143.73, 141.36, 139.87, 135.23, 135.18, 135.13, 134.10, 130.67, 129.01, 128.25, 128.21, 128.17, 127.90, 127.27, 125.89, 125.30, 120.09, 118.72, 87.59, 77.42, 77.16, 76.91, 67.56, 53.36, 47.10, 45.05, 42.92, 29.50, 28.53, 24.96, 19.42, 18.07, 12.49.

# LC-MS traces of analyte and internal standard

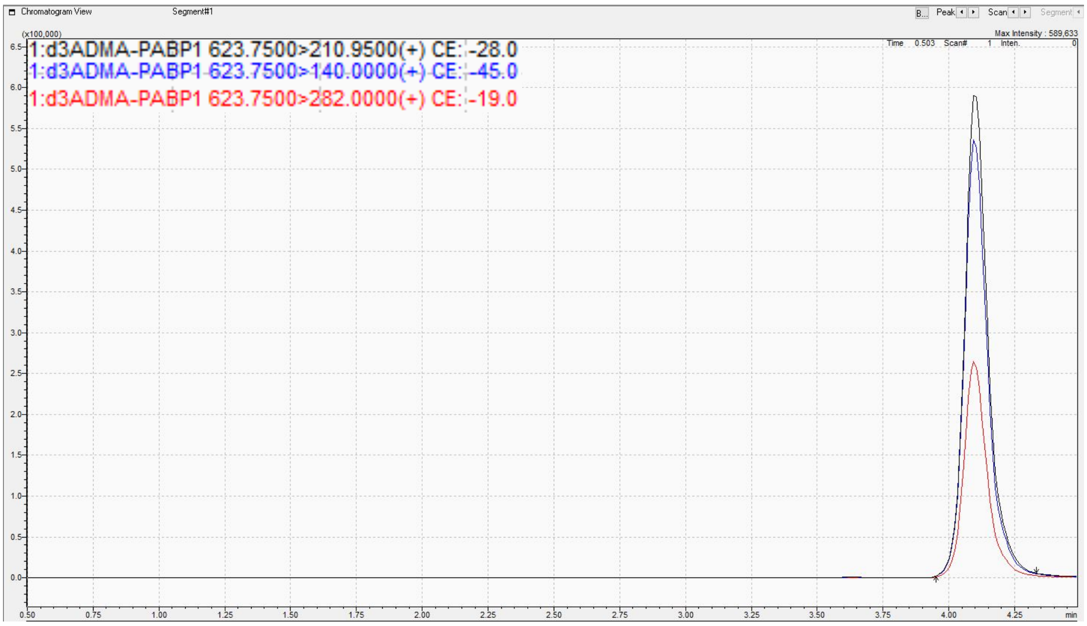


**Figure S1.** LC-MS/MS traces of PABP1$^{456\text{-}466}$R$^{460}$-$d_6$-aDMA (internal standard). MS method: 0.5-4.5 min; retention time 4.101 min, Q1 mass detection; 623.75; Q3 mass detection: 282.00, 210.95, 140.00.

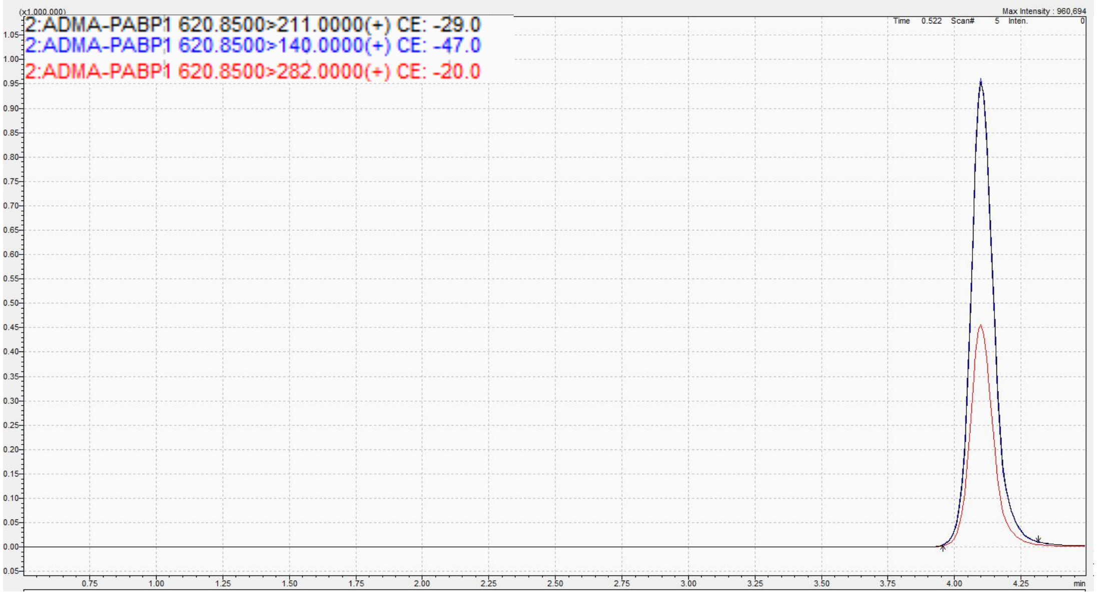


**Figure S2.** LC-MS/MS traces of PABP1$^{456\text{-}466}$ R$^{460}$-aDMA (methylated product). MS method: 0.5-4.5 min; retention time 4.101 min, Q1 mass detection; 620.85; Q3 mass detection: 282.00, 211.00, 140.00.

# Kinetic analysis of CARM1 substrates

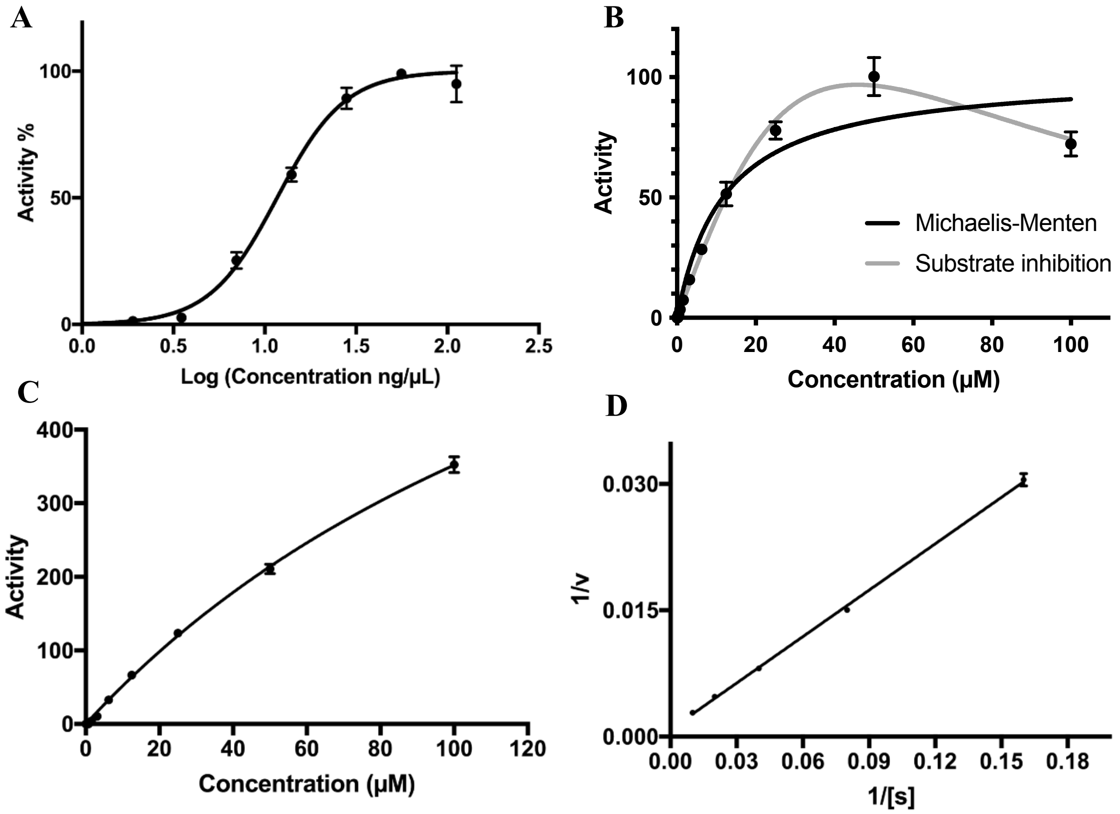


**Figure S3. A)** $EC_{50}$ curve for CARM1, $EC_{50}$ = 11.68 ± 0.33 ng/µL. **B)** Substrate inhibition plot and Michaelis-Menten Plot for $K_M$ value determination of PABP1$^{456\text{-}466}$, $K_M^{app}{}_{,\text{ PABP1 456-466}}$= 12.03 ± 2.28 µM. **C)** Michaelis-Menten Plot and Lineweaver Burk plot. **D)** for $K_M$ value determination of AdoMet, $K_{M,\text{ AdoMet}}$= 5.46 ± 0.01 µM.

## $IC_{50}$ curves for compounds 1-9

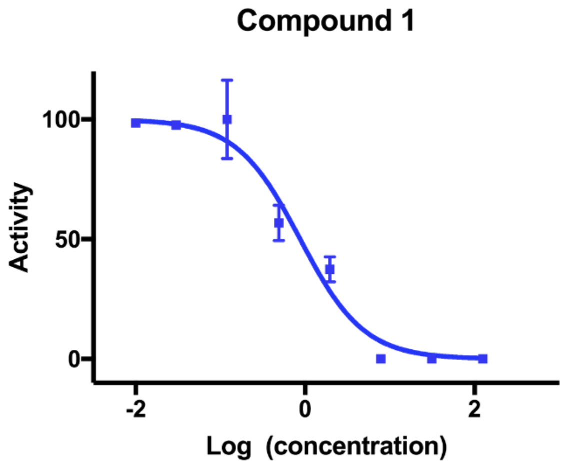


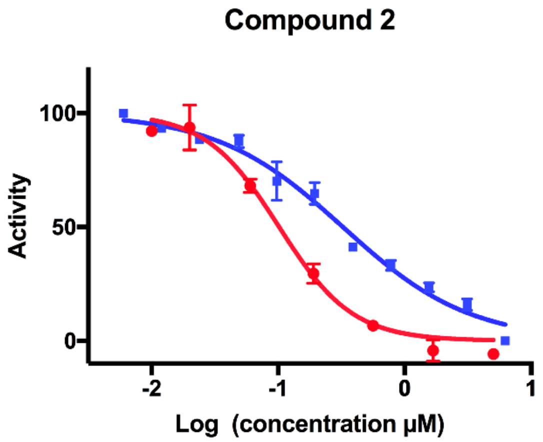


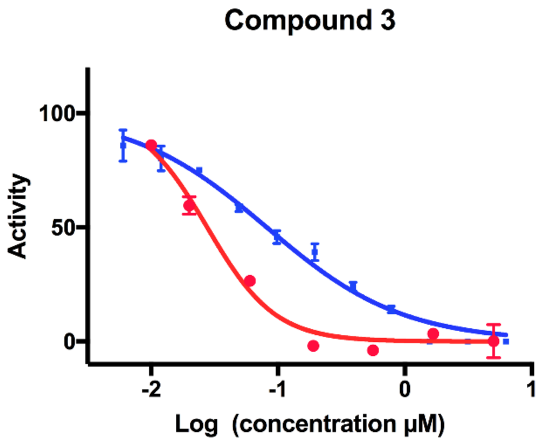


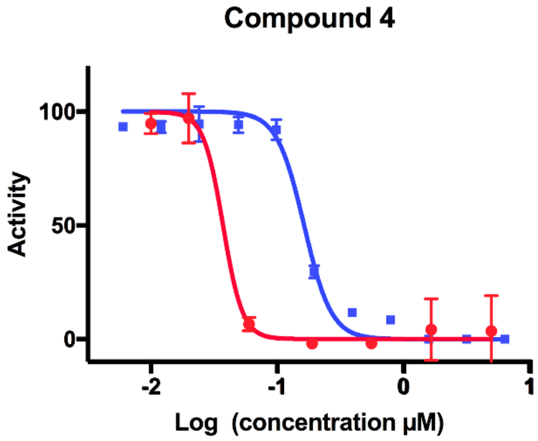


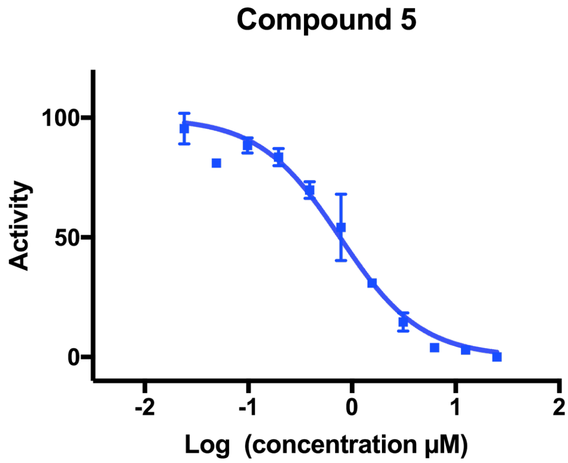


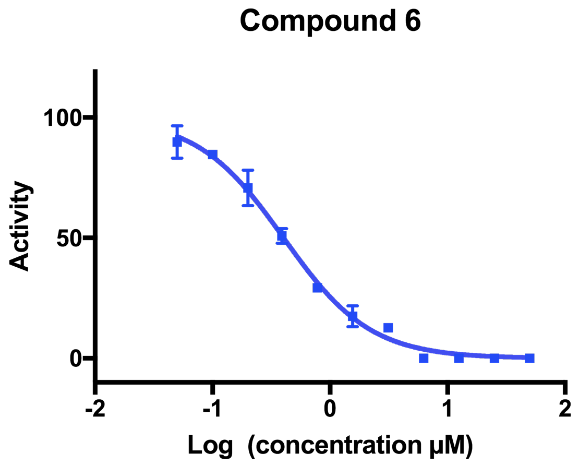

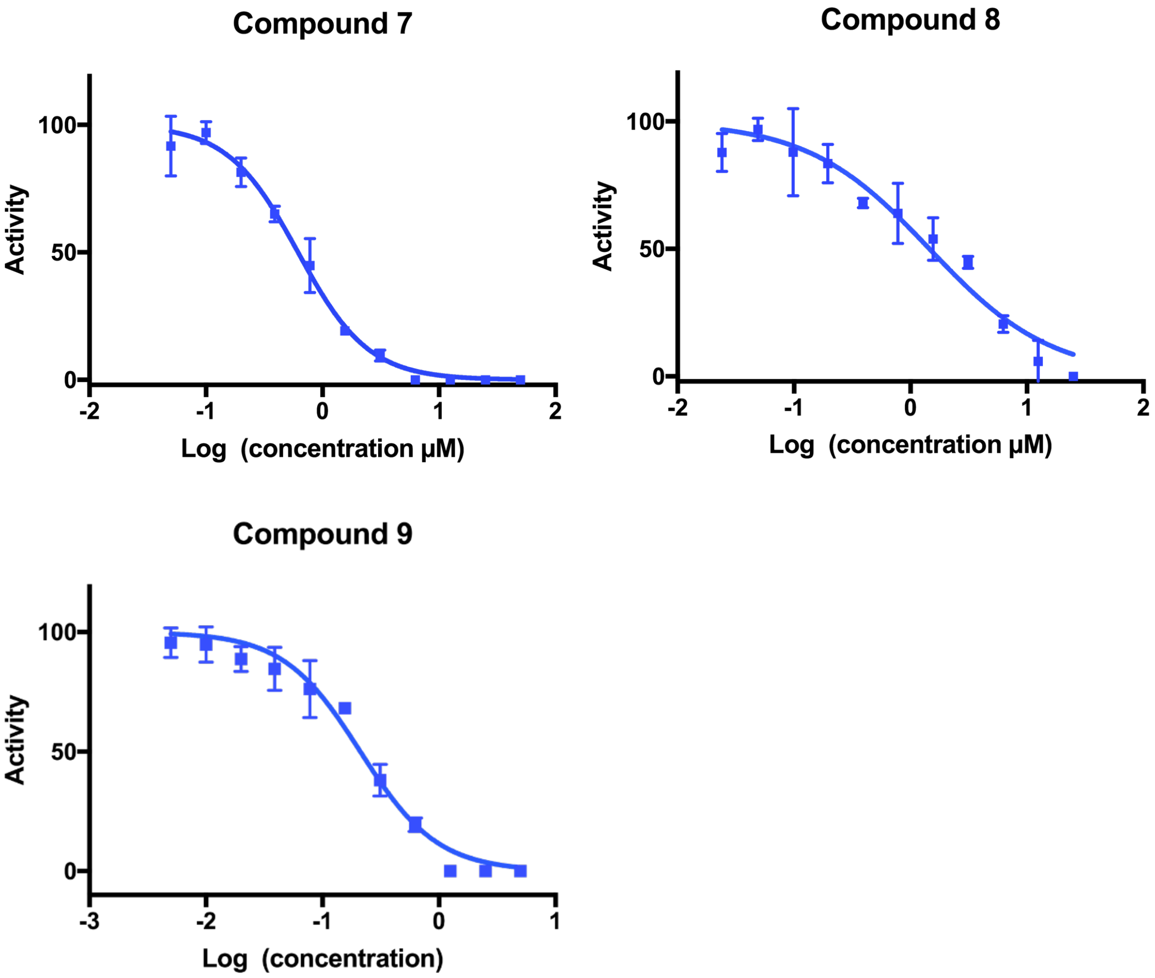


**Figure S4.** $IC_{50}$ curves of inhibitors **1**-**9**. Blue curves represent the measurements obtained using the MRM LC-MS assay and red curves correspond to measurements obtained using commercially available the ELISA assay kit. The ELISA assay based $IC_{50}$ values for compounds **1**, **5-9** presented in Table 3 of the manuscript are taken from our previously published work and the corresponding inhibition curves can be found there (refs 27 and 20).

**HPLC and High Resolution Mass Spectrometry data for PABP1$^{456\text{-}466}$ peptides**

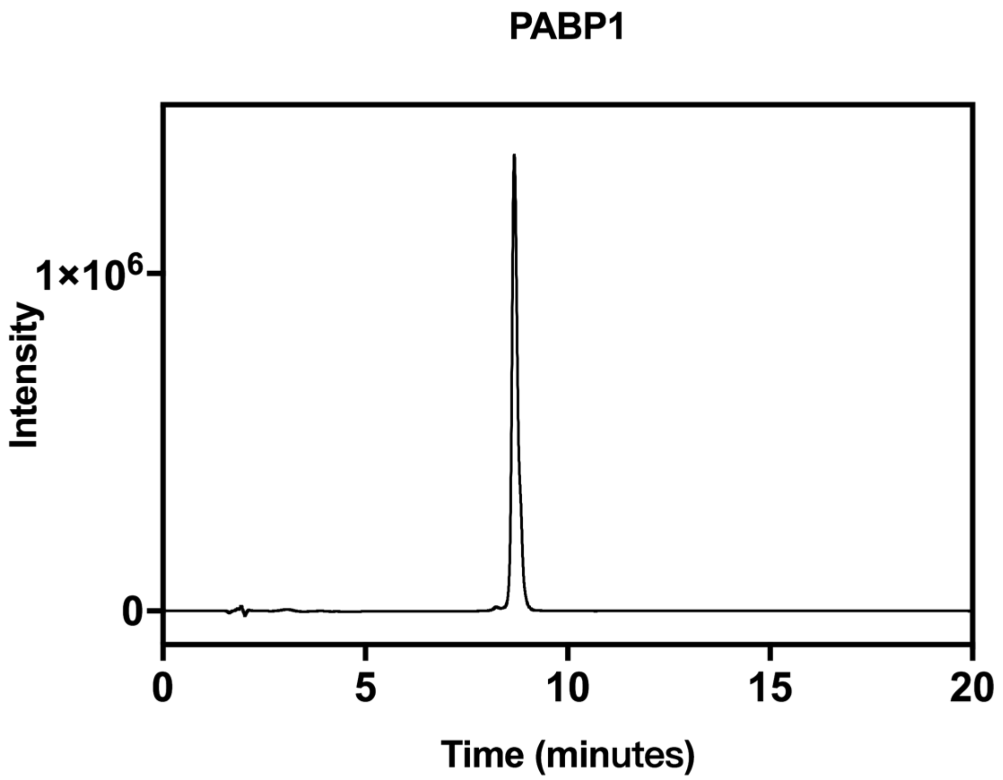


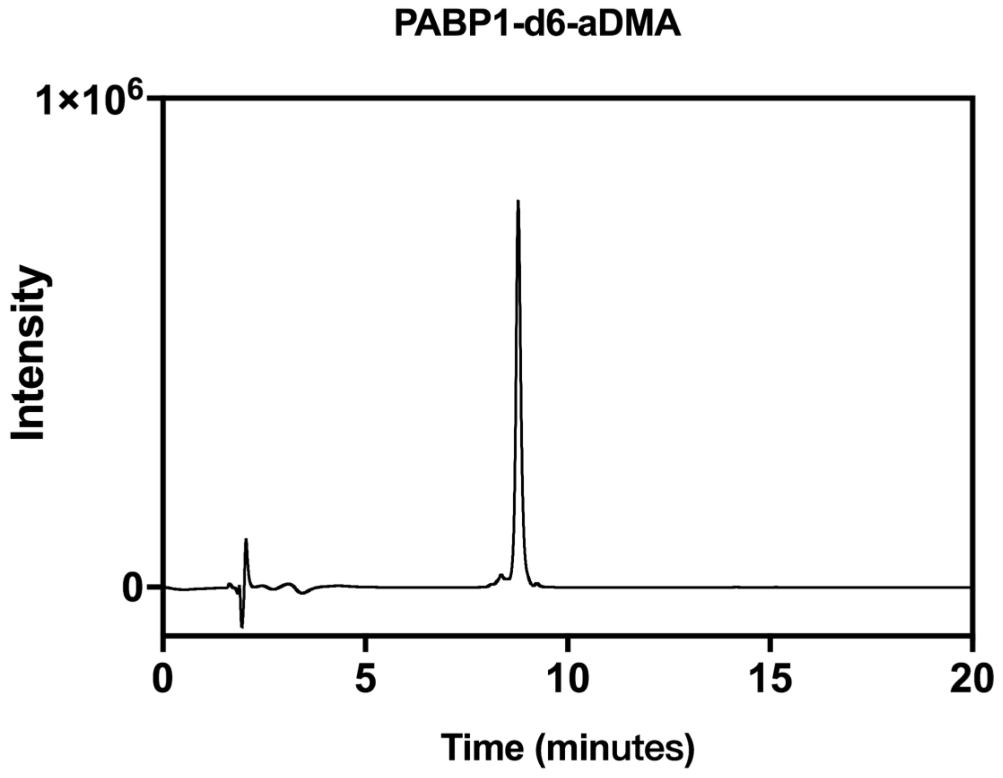

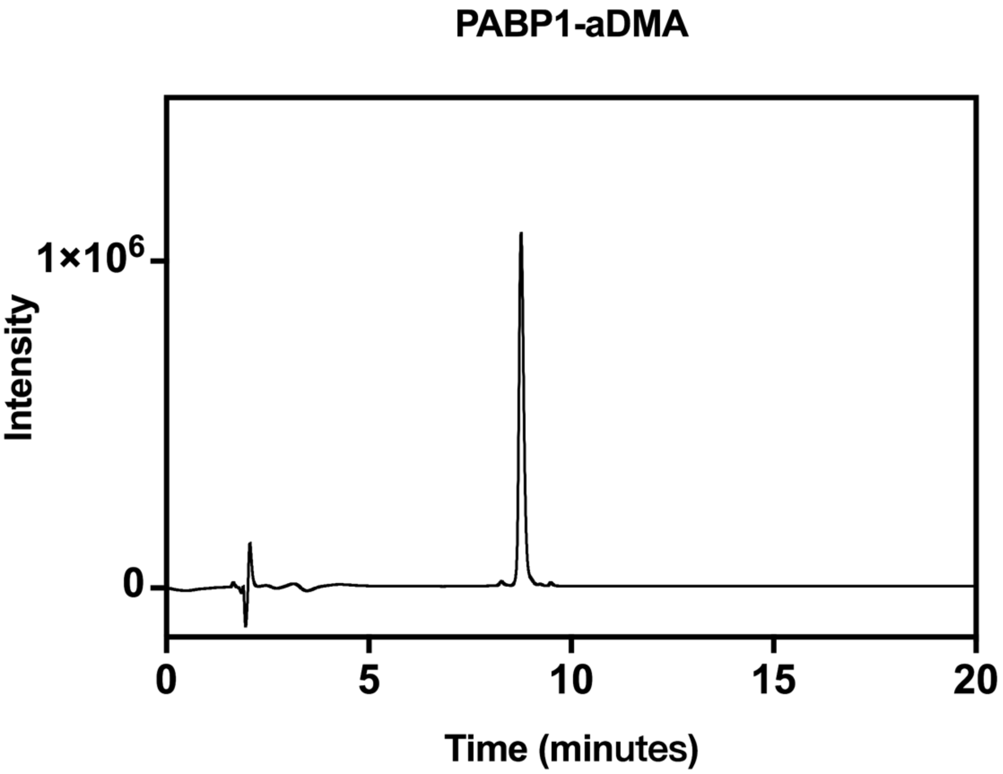


## High Resolution Mass Spectrometry (HRMS)

**PABP1$^{456\text{-}466}$** (m/z):
$[M+H]^+$ calculated for $C_{55}H_{86}N_{15}O_{14}S^+$, 1212.6199, found 1212.6206.

**PABP1$^{456\text{-}466}$R$^{460}$-aDMA** (m/z):
$[M+H]^+$ calculated for $C_{57}H_{90}N_{15}O_{14}S^+$, 1240.6512, found 1240.6516.

**PABP1$^{456\text{-}466}$R$^{460}$-*$d_6$*-aDMA** (m/z):
$[M+H]^+$ calculated for $C_{57}H_{84}D_6N_{15}O_{14}S^+$, 1246.6889, found 1246.6894

# NMR Data of Fmoc-$d_6$-aDMA-OH

## $^{1}H$ NMR (500 MHz, $CDCl_3$)

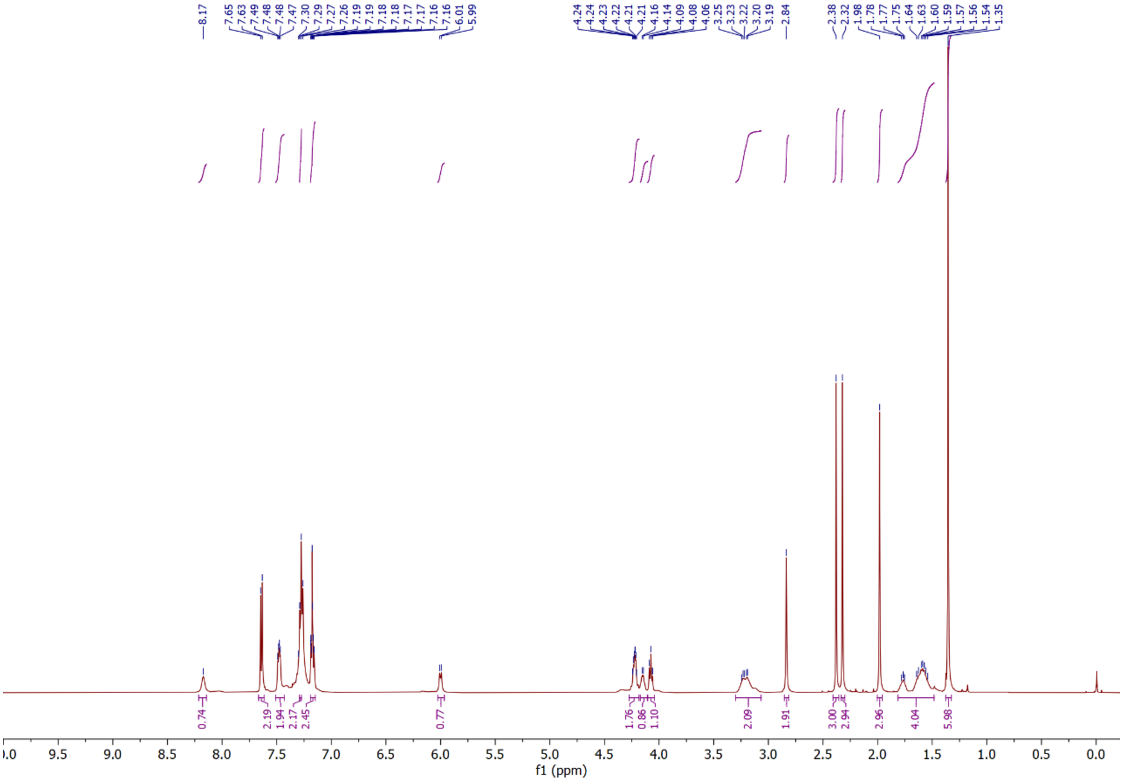

## $^{13}C$ NMR (126 MHz, $CDCl_3$)

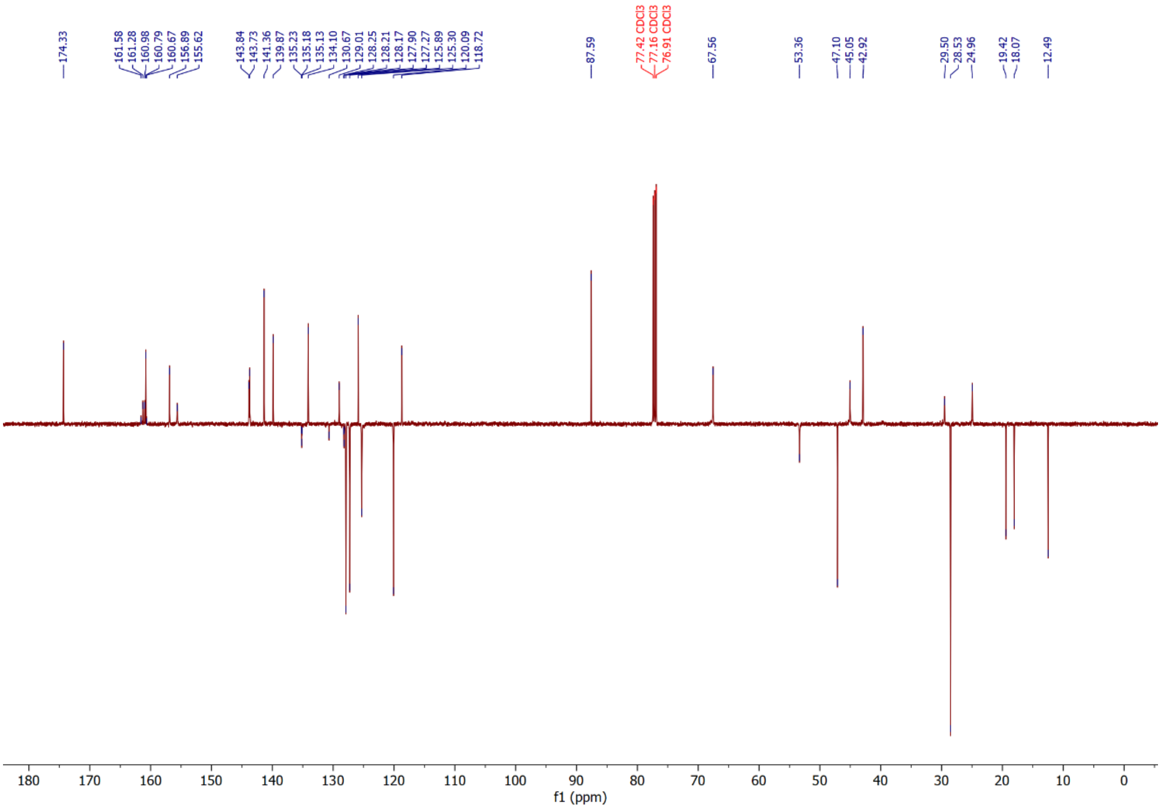

## References

(1) Martin, N. I., and Liskamp, R. M. J. Preparation of NG-Substituted l-Arginine Analogues Suitable for Solid Phase Peptide Synthesis. *The Journal of Organic Chemistry* **2008,** *73*, 7849-7851.
